# Silicon Affects Root Development, Tissue Mineral Content, and Expression of Silicon Transporter Genes in Poinsettia (*Euphorbia pulcherrima* Willd.) Cultivars

**DOI:** 10.3390/plants8060180

**Published:** 2019-06-17

**Authors:** Jiangtao Hu, Xuan Cai, Byoung Ryong Jeong

**Affiliations:** 1 Department of Horticulture, Division of Applied Life Science (BK21 Plus Program), Graduate School of Gyeongsang National University, Jinju 52828, Korea; hujiangtao@gnu.ac.kr; 2 Institute of Agriculture and Life Science, Gyeongsang National University, Jinju 52828, Korea; luckilyxuan@gmail.com; 3 Research Institute of Life Science, Gyeongsang National University, Jinju 52828, Korea

**Keywords:** cutting propagation, mineral content, poinsettia, root development, silicon transporters

## Abstract

The effects of silicon (Si) on root development, mineral content, and expression of Si transporter genes in *Euphorbia pulcherrima* Willd. ‘Flame’, ‘Mable Bell’, ‘Green Star’, ‘Pink Bell’, and ‘Peach Bowl’ cultivars were investigated in this study. Stem cuttings in a propagation bench were drenched regularly with a solution containing either 0 (control) or 50 ppm of silicon (Si treatment) from potassium silicate (K_2_SiO_3_), with a 25 °C mean air temperature and 80% relative humidity (RH) under 70% shading. The results showed that the ‘Flame’ treated with Si had a significantly higher survival ratio as compared with that of the control (*P* ≤ 0.05) and that the Si treatment improved number of roots, length of longest root, fresh root weight, and dry root weight in all cultivars except ‘Mable Bell’. Supplementary Si increased the content of magnesium (Mg) and decreased the content of boron (B) and zinc (Zn) in the roots. The content of sulfur (S) in the shoots was increased by supplementary Si. The relative expression of *Lsi1* and *Lsi2* was higher in ‘Peach Bowl’, while it was lower in ‘Mable Bell’ and ‘Green Star’, which may be caused by the differing accumulation of Si in the shoot. Overall, supplementary Si had beneficial effects during cutting propagation of poinsettia cultivars, although these effects were cultivar-dependent.

## 1. Introduction

Poinsettia (*Euphorbia pulcherrima* Willd.) is one of the most famous ornamental plants for its colored bracts. The attractive bracts can remain fresh during the winter season, which makes it popular as a Christmas decoration as well as a general indoor decoration. In addition to its ornamental value, the latex from poinsettia can be used as a veterinary medicine for livestock to kill maggots [1].

In commercial production, poinsettia is propagated from stem cuttings. It is common for the harvested cuttings to be stored and transported to growers. Delays in this process will negatively impact the quality of the cuttings and therefore, the subsequent propagation performance [2]. A study suggests that poinsettia ‘Prestige’ can be stored for 8 to 10 days under optimal temperatures (10–15 °C). However, the storage potential decreases under non-optimal temperatures [3]. Thus, multiple strategies should be determined to improve the rooting capacity and propagation performance of poinsettia. The rooting capacity is essential to the survival of cuttings, which can be indicated by the number of roots. And propagation performance can be indicated by the length of roots, fresh root weight, and dry root weight. Nowadays, growth regulators such as salicylic acid [4], indole 3-butyric acid [5], indole-3-acetic acid, and naphthalene acetic acid [6] have been used in the propagation of poinsettia. In addition, supplementary phosphorus can lead to optimum yield and quality of poinsettia, which suggests that nutrient application might be an alternative method to improve poinsettia propagation [7].

Silicon (Si) is largely present in the soil. However, only soluble silicon oxide in the form of monosilicic acid [Si(OH)_4_] can be absorbed by plants. As high-tech greenhouse production practices are developed and adopted, soil is substituted by commercial substrates. This substitution from soil to commercial substrates signifies a limited Si amount available for plants. On the other hand, proteins encoded by *low silicon 1 (Lsi1*) and *low silicon 2* (*Lsi2*) genes absorb Si from the substrate and transport it to the xylem [8]. The suppression or lack of *Lsi1* in plants will result in either reduced Si uptake or low Si accumulation [9,10]. Analysis of genome and transcriptome data shows that Lsi1 member has two conserved asparagine–proline–alanine (NPA) domains and an aromatic/arginine (ar/R) selectivity filter. Plants can be divided into low, moderate, and high Si accumulators based on the ar/R selectivity filter [11]. Furthermore, a precise distance of 108 amino acids (AA) between the NPA domains is reported to be necessary for Si uptake. The Lsi1 mutated into NPA domains with 109- or 107- AA spacing will fail to absorb the Si [10]. Thus, different cultivars may have varying abilities to uptake Si due to the different efficiencies of Lsi1 and Lsi2. Moreover, Si was reported to be a non-essential element for several crops [12]. As information on supplementary Si in poinsettia is very limited, it is helpful to investigate the role of Si in different poinsettia cultivars during cutting propagation.

Our previous studies have revealed the beneficial effects of Si on rooting [13], adventitious shoot regeneration [14], and abiotic stress mitigation [15,16,17,18] in different plants. In this study, the effect of Si on root development and the most frequently-studied mineral elements were investigated. Moreover, the expression of *Lsi1* and *Lsi2* was determined as they play essential roles in absorbing and transporting Si. Overall, we supplied five poinsettia cultivars (‘Flame’, ‘Mable Bell’, ‘Green Star’, ‘Pink Bell’, and ‘Peach Bowl’) with Si during the cutting propagation stage, aiming to obtain information on how Si affects the cutting propagation of these cultivars.

## 2. Materials and Methods

### 2.1. Plant Materials and Growth Conditions

Terminal cuttings of *Euphorbia pulcherrima* Willd. ‘Flame’, ‘Mable Bell’, ‘Green Star’, ‘Pink Bell’, and ‘Peach Bowl’ were collected from the Rural Development Administration (RDA) of the Republic of Korea. The cuttings were harvested and transported to the experimental site in June within a time span of one day. The cuttings were then grown in foam wedge substrate in trays (Smithers Oasis Korea, Seoul, Korea) and kept on a fogged (fogged for 10 min every 15 min) propagation bench with a mean air temperature of 25 °C and relative humidity of 80% for four weeks in a glasshouse at Gyeongsang National University, Jinju, Korea. To avoid differences caused by original materials and location of trays on the bench, cuttings were randomly selected for each treatment, and the location of trays was separated by cultivar, treatment, and replicate.

### 2.2. Supplementary Si Treatments

Cuttings in trays were drenched with a nutrient solution containing either 0 (control) or 50 ppm of Si (Si treatment) from potassium silicate (K_2_SiO_3_) until the substrate was thoroughly soaked. The solutions were applied every three days for four weeks. The additional potassium introduced by K_2_SiO_3_ was balanced with reduced KNO_3_, and the resultant nitrate loss was compensated with nitric acid. In details, the composition of the nutrient solution for the control (the control solution) was as follows (in ppm): 708.00 Ca(NO_3_)_2_·4H_2_O, 246.00 MgSO_4_·7H_2_O, 505.00 KNO_3_, 230.00 NH_4_H_2_PO_4_, 1.24 H_3_BO_3_, 0.12 CuSO_4_·5H_2_O, 4.00 Fe-EDTA, 2.20 MnSO_4_·4H_2_O, 0.08 H_2_MoO_4_, and 1.15 ZnSO_4_·7H_2_O. The composition of the nutrient solution for the Si treatment (the Si solution) was as follows (in ppm): 50.00 K_2_SiO_3_, 708.00 Ca(NO_3_)_2_·4H_2_O, 246.00 MgSO_4_·7H_2_O, 439.51 KNO_3_, 230.00 NH_4_H_2_PO_4_, 1.24 H_3_BO_3_, 0.12 CuSO_4_·5H_2_O, 4.00 Fe-EDTA, 2.20 MnSO_4_·4H_2_O, 0.08 H_2_MoO_4_, 1.15 ZnSO_4_·7H_2_O, and 40.80 HNO_3_. The pH of final solutions (both the control and Si treatment) was adjusted to 6.0 using 1 mol·L^−1^ hydrochloric acid. Each treatment consisted of three replicates, and each replicate contained 15 cuttings.

### 2.3. Measurement of Root Development and Sampling

The foam wedge substrate was carefully removed to expose the roots. After counting the number of roots and measuring the length of the longest root, the roots were removed for measurement of fresh weight. Shoot and root samples were immersed in liquid nitrogen and stored at −80 °C for gene expression analysis. Root dry weight was measured after drying them in an oven at 65 °C for three days. The dried root samples were further used to determine tissue mineral contents.

### 2.4. Determination of Tissue Mineral Content

Analysis of tissue mineral content was made after four weeks of cultivation. To determine the content of silicon (Si), boron (B), copper (Cu), iron (Fe), manganese (Mn), zinc (Zn), potassium (K), calcium (Ca), magnesium (Mg), phosphorus (P), and sulfur (S), dried root or shoot samples from each treatment were mixed together and ground with a stainless mill (Cytclotec, Model 1093, Tector, Hoganas, Sweden). The samples were ashed at 525 °C for 4 h in a Nabertherm muffle furnace (Model LV 5/11/B180, Lilienthal, Bremen, Germany). The ash was dissolved in 5 mL 25% HCl, followed by dilution with 20 mL of warm distilled water. The content was then measured three times for each treatment using an inductively coupled plasma (ICP) spectrometer (Optima 4300DV/5300DV, Perkin Elmer, Germany).

### 2.5. Quantitative RT-PCR

Roots were obtained from poinsettia plants cultivated for four weeks. Total RNA was extracted using the Easy-Spin Total RNA Extraction Kit (iNtRON Biotechnology, Seoul, Korea) and then reverse transcribed to cDNA using the PrimeScript RT Reagent Kit (Takara, Shiga, Japan). Three biological replicates and two technical replicates were adopted for each treatment. The reactions were carried out on the Rotor-Gene Q detection system (Qiagen, Hilden, Germany) machine. The primers used in this study are as follows: *Lsi1*: 5′-GGCTCTACGGTTCTCCTG-3′ (forward), 5′-CCTGCTTGTGCTCCTAAT-3′ (reverse). *Lsi2*: 5′-CCGTCCAGTAGGGTAGAGT-3′ (forward), 5′-ATGCCAATGGTTACAAGGT-3′ (reverse). 18S (reference gene): 5′-ATGATAACTCGACGGATCGC-3′ (forward), 5′-CTTGGATGTGGTAGCCGT-3′ (reverse). The relative gene expressions were calculated using the 2^−ΔΔCt^ method.

### 2.6. Statistical Analysis

The data analysis was performed with the SAS statistical software Release 8.2 (SAS Inst., Cary. N.C., USA). The differences between the control and the Si treatment were tested by Student’s *t*-test (*P* ≤ 0.05). Moreover, data from the quantitative RT-PCR results were analyzed by Duncan’s test (*P* ≤ 0.05).

## 3. Results

### 3.1. Survival Ratio

**Figure 1:**
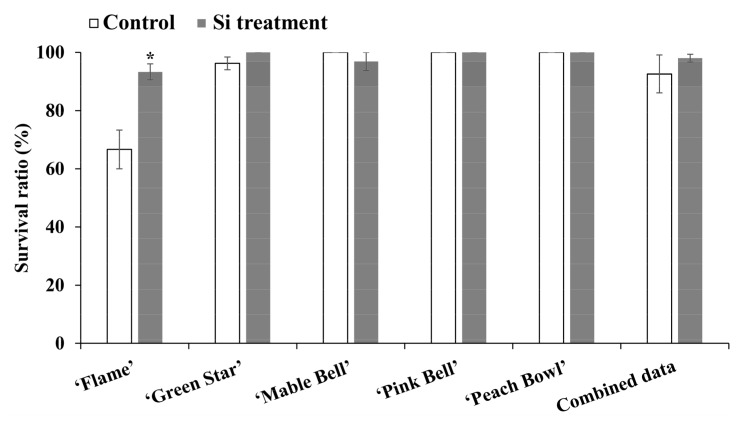
Survival ratio of the poinsettia cuttings. Data were represented by the mean ± SE (bars). Combined data is the average value of all the cultivars. Asterisks represent significant differences between the control and the Si treatment according to Student’s *t*-test (*P* ≤ 0.05).

The cuttings went through stresses caused by the delay in transit and high temperatures. As a result, some of the cuttings rotted at the base of the stem after cultivating for several days. Without Si supplementation, the ‘Flame’ cuttings had a survival ratio of 66.7%, whereas the survival ratio of ‘Flame’ cuttings treated with Si was 93.3%. Supplementary Si did not significantly affect the survival ratio of the other cultivars. ‘Green Star’ treated with Si, ‘Mable Bell’ in the control, as well as the control and Si-treated ‘Pink Bell’ and ‘Peach Bowl’ had survival ratio of 100% (Figure 1).

### 3.2. Root Development

**Figure 2:**
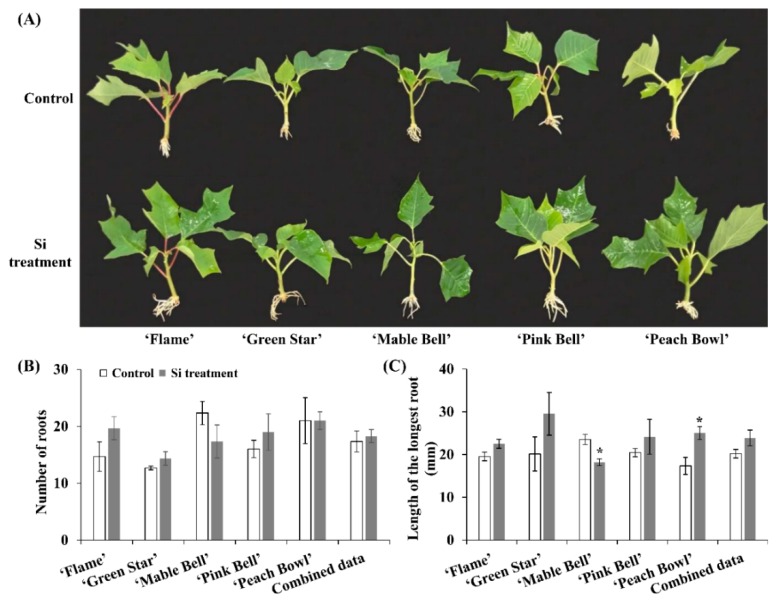

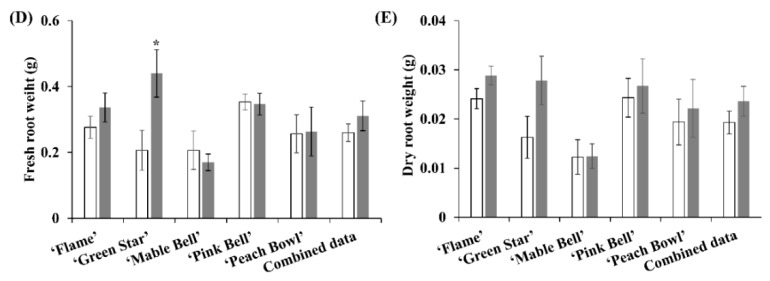
The root development (**A**), number of roots (**B**), length of the longest root (**C**), fresh root weight (**D**), and dry root weight (**E**) of the control and Si-treated poinsettia cultivars. Data were represented by the mean ± SE (bars). Combined data is the average value of all the cultivars. Asterisks represent significant differences between control and Si treatment according to Student’s *t*-test (*P* ≤ 0.05).

The roots were fully developed after four weeks of cultivation (Figure 2A). Except in the case of the length of the longest root for ‘Mable Bell’ and ‘Peach Bowl’ and fresh root weight for ‘Green Star’, the Si treatment induced no statistical differences in the root development of different poinsettia cultivars (Figure 2B–E). However, number of roots, length of the longest root, fresh root weight, and dry root weight in poinsettia cultivars treated with Si were greater than those of the control except for ‘Mable Bell’. Moreover, the combined data (average value of all the cultivars) showed that Si promoted root development in poinsettia, whereas there is no significant difference between the control and the Si treatment.

### 3.3. Tissue Mineral Content

Mineral analyses were conducted for both the shoots and roots of poinsettia cultivars (Table 1; Table 2). In the control groups, ‘Mable Bell’ had the highest accumulation of Si in both the shoot and root, followed by that of ‘Green Star’, ‘Flame’, ‘Pink Bell’, and ‘Pink Bowl’. Moreover, the Si, K, Ca, and Mg levels in the root of ‘Flame’ were relatively lower than other cultivars. As a result of the Si supplementation, the Si content was elevated in the roots of ‘Flame’, ‘Green Star’, and ‘Peach Bowl’ and in the shoots of ‘Flame’, ‘Green Star’, ‘Pink Bell’, and ‘Peach Bowl’, whereas it was reduced in the roots of ‘Mable Bell’ and ‘Pink Bell’ and in the shoot of ‘Mable Bell’. Along with the increased Si levels in the roots of poinsettia, Mg was significantly increased, and levels of micronutrients, such as B and Zn, were decreased. However, in the shoots, the Si treatment resulted in an increased S. Moreover, the *F*-test results showed that Cu was not significantly affected by supplementary Si in the root and shoot, while levels of B, Fe, Mn, Zn, K, Mg, and S were significantly influenced by supplementary Si.

### 3.4. Expression of Si Transporter Genes

**Figure 3:**
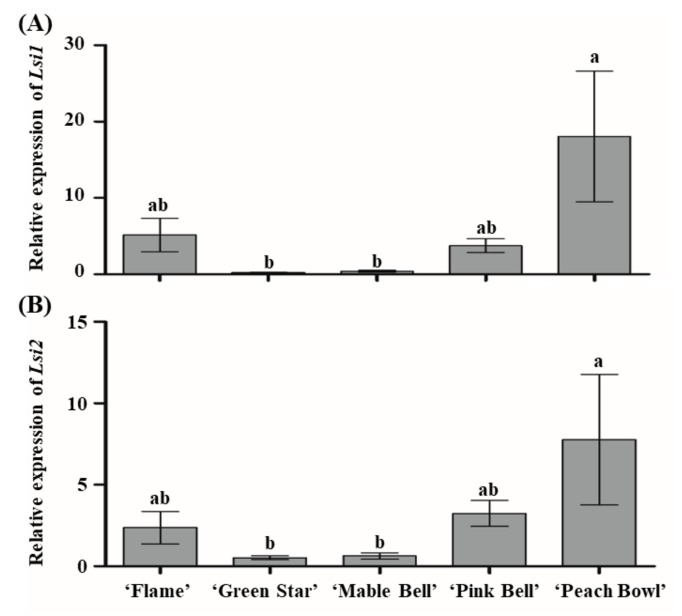
Relative expression of *Lsi1* (**A**) and *Lsi2* (**B**) in roots of poinsettia cultivars. Data were represented by the mean ± SE (bars). The letters represent significant differences between the control and the Si treatment, according to Duncan’s test (*P* ≤ 0.05).

As Si accumulation is significantly different among the cultivars, even without Si supplementation, and as Si uptake is affected by Si transporter genes, the expression of *Lsi1* and *Lsi2* was investigated in the roots of the poinsettia cultivars. The results illustrated that the expression of Si transporter genes was greatly different among the poinsettia cultivars (Figure 3A,B). Both *Lsi1* and *Lsi2* were highly expressed in ‘Peach Bowl’, while they were lowly expressed in ‘Mable Bell’ and ‘Green Star’.

## 4. Discussion

Cutting propagation using terminal cuttings from stock plants is widely used in floriculture. The unrooted cuttings mainly use nutrients maintained in the tissue for root formation. Thus, it is particularly important to replenish fertilizers once the root emerges. However, a study has proven that early fertilization is also important during cutting propagation [19]. Moreover, petunia cuttings were found to be able to uptake both macronutrients and micronutrients through the stem before roots initiate, improving the root development as a result [20]. Generally, root initiation and development in poinsettia will take 10 to 14 days. Thus, supplementary Si, during this phase, has the potential to reduce abiotic stresses and/or promote root development. The results of this study showed that Si supplementation significantly enhances the survival ratio of ‘Flame’ cuttings. Si has been reported to decrease transpiration in crops, such as maize [21], cucumber [22], and rice [23]. It is speculated that Si protected poinsettia by reducing the transpiration during cutting propagation. Notably, the survival ratio of ‘Flame’ in the control group is significantly lower as compared with other cultivars (Figure 1). As the cuttings were randomly selected, the location of trays was separately arranged, and the root development of ‘Flame’ was enhanced in the Si treatment (Figure 2), ‘Flame’ is supposed to be more susceptible to the Si treatment than other cultivars.

Root formation and development are essential to cutting propagation of poinsettia. Cuttings can be moved out early from fogging systems if they have well-developed roots, which can reduce the risk of diseases caused by the high humidity. Our previous studies have shown that root biomass was increased by silicate fertilization in kalanchoe and carnation [13]. Mist application of sodium silicate was also shown to increase rooting in roses [24]. Similarly, number of roots, length of longest root, fresh root weight, and dry root weight were improved by Si supplementation in all cultivars except ‘Mable Bell’ in this study. It was observed that Si accumulation in the root and shoot of ‘Mable Bell’ decreased following Si supplementation, which may be the reason for these results. There is no information on the mechanisms through which Si affects rooting. Nevertheless, decreased transpiration following Si application may be one of the reasons for the general improvement in rooting, as antitranspirants have been reported to increase rooting in crops [25].

Supplementary Si affects the uptake of minerals, such as P, Mn, K, N (nitrogen), and Ca [26]. It can also alleviate toxicity of heavy metals, such as Zn (zinc), Mn, Al (aluminum), Cu, and Cd (cadmium), under heavy metal stress [27]. We also found that accumulation of minerals, such as B, Fe, Mn, Zn, K, Mg, and S, was changed by supplementary Si. However, some of the mineral elements did not show significant changes, which may be caused by the less abiotic stress after four weeks of cultivation or the short-time cultivation. For instance, a study has demonstrated that Si had no effects on the mineral uptake under normal conditions. However, mineral contents were significantly decreased by supplementary Si for plants under water stress [23]. Another study showed that strawberry plants in the control nutrient solution accumulated only 0.4% Si (in terms of dry weight) after one month of cultivation, while it increased to 3.0% after fertilization with Si for three months [28]. Moreover, the content of S was increased by supplementary Si in poinsettia shoots, which is consistent with the results from a study of gerbera [29]. As shown in Table 1, the root in control plants of ‘Flame’ has lower levels of Si, K, Ca, Mg, and Mn. However, levels of other mineral elements including P, S, B, Fe, Zn are normal. As all of the mineral elements were measured from the same processed sample, it could not be a problem caused by the original sampling. On the other hand, the levels of Si, K, Ca, and Mg are enhanced by the Si treatment in ‘Flame’. Therefore, Si is supposed to be essential for ‘Flame’. Thus, this nutritional problem may be caused by a shortage of Si.

Higher expression of *Lsi1* and *Lsi2* results in greater Si uptake in rice [30]. Interestingly, we found that the expression of *Lsi1* and *Lsi2* and Si accumulation were inversely related in both the root and shoot. A similar result was found in rice. The expression of Si transporter genes was reduced by Si accumulation in the shoot [31]. These authors further found that different pumpkin cultivars had allelic variations in one amino acid residue of Lsi1, which resulted in differing Si uptakes [32]. As shown in Table 2, a reduction to a third of Si content for ‘Mable Bell’ and an approximate doubling of Si content for ‘Flame’ are found in the shoot. We can also found that ‘Mable Bell’ has the highest accumulation of Si in the control groups, which may result in a lower expression of *Lsi1* and *Lsi2* (Figure 3). Although *Lsi1* and *Lsi2* are highly expressed in both ‘Flame’ and ‘Peach Bowl’, the increase of Si content in ‘Flame’ is greater than that in ‘Peach Bowl’. It is speculated that there may have allelic variations in the Lsi1 between ‘Flame’ and ‘Peach Bowl’, and as a result, the capacities of Si uptake of both cultivars are different. Thus, the dramatically increased Si content in ‘Flame’ and the reduced Si content in the roots of ‘Mable Bell’ and ‘Pink Bell’ and in the shoot of ‘Mable Bell’ may be explained by the different Si uptake capacities and/or the different expressions of Si transporter genes among the poinsettia cultivars. The reasons for how supplementary Si differently affects different poinsettia cultivars during cutting propagation requires further investigation to be clearly explained.

## 5. Conclusions

Supplementary Si has the potential to improve the survival ratio of poinsettia during cutting propagation. Root development characteristics, including the number of roots, length of the longest root, fresh root weight, and dry root weight were enhanced by Si supplementation, possibly because of silicon’s role in reducing transpiration. Supplementary Si during cutting propagation also changed the mineral uptake in poinsettia. However, these changes vary among the different cultivars. *Lsi1* and *Lsi2* expressions and Si accumulation were shown to be inversely related in the roots of poinsettia. In addition, Si accumulation significantly varied among the poinsettia cultivars in this study. Thus, more academic research is required to explain these results.

## Figures and Tables

**Table 1:** Mineral content in the roots of the poinsettia cultivars.

Cultivar (A)	Si (mg·L^−1^) (B)	Si (mg·g^−1^ Dry Weight)	Macronutrients (mg·g^−1^ Dry Weight)					Micronutrients (µg·g^−1^ Dry Weight)				
			K	Ca	Mg	P	S	B	Cu	Fe	Mn	Zn
‘Flame’	0	0.32	9.74	1.65	0.40	1.04	0.84	63.19	28.08	48.69	0.69	94.50
	50	0.47^*z^	14.01^*^	2.71^*^	0.65^*^	1.51^*^	0.77^*^	49.65^*^	42.15^*^	47.54	5.20^*^	41.28^*^
‘Green Star’	0	0.58	15.43	3.92	0.80	2.10	1.31	75.31	46.62	64.04	17.76	40.13
	50	0.68^*^	13.29^*^	3.03^*^	0.84^*^	1.80^*^	1.15^*^	40.92^*^	39.12^*^	95.81^*^	7.58^*^	28.24^*^
‘Mable Bell’	0	0.78	13.03	3.96	0.72	1.26	1.21	92.62	62.05	81.52	3.17	35.55
	50	0.59^*^	13.07	3.30^*^	0.61^*^	1.63^*^	1.56^*^	84.00	55.85^*^	81.99	0.89^*^	58.98^*^
‘Pink Bell’	0	0.26	11.56	2.58	0.85	1.82	1.20	36.19	36.42	47.03	2.40	36.53
	50	0.22^*^	9.75^*^	2.81^*^	0.90^*^	1.47^*^	0.98^*^	28.26^*^	32.32^*^	49.36^*^	4.68^*^	21.30^*^
‘Peach Bowl’	0	0.23	9.83	2.74	0.70	1.93	1.11	29.32	30.95	44.74	1.88	46.87
	50	0.36^*^	10.45^*^	3.14^*^	0.78^*^	1.67^*^	1.10	21.76	34.08^*^	58.71^*^	6.01^*^	29.93^*^
*F*-test	A	***^y^	***	***	***	***	***	***	***	***	***	***
	B	***	**	NS	***	NS	**	***	NS	***	***	***
	A×B	***	***	***	***	***	***	***	***	***	***	***

Data were represented by the mean. Each treatment was measured three times. ^z^Asterisks represent significant differences between the control and the Si treatment according to Student’s *t*-test (*P* ≤ 0.05). ^y^NS, *, **, *** Non-significant or significant at *P* ≤ 0.05, 0.01, 0.001, respectively.

**Table 2:** Mineral content in the shoots of the poinsettia cultivars.

Cultivar(A)	Si (mg·L^−1^) (B)	Si (mg·g^−1^ dry weight)	Macronutrients (mg·g^−1^ Dry Weight)					Micronutrients (µg·g^−1^ Dry Weight)				
			K	Ca	Mg	P	S	B	Cu	Fe	Mn	Zn
‘Flame’	0	0.10	6.19	2.69	1.00	1.78	0.39	14.42	3.15	25.89	12.88	21.01
	50	0.26^*z^	6.66^*^	2.61	1.00	1.68^*^	0.49^*^	11.68^*^	4.06^*^	30.13^*^	10.33^*^	16.34^*^
‘Green Star’	0	0.18	8.21	2.81	1.33	2.89	0.63	19.19	6.71	38.68	19.53	22.93
	50	0.19^*^	7.09^*^	3.58^*^	1.61^*^	2.25^*^	0.73^*^	17.05^*^	5.20^*^	37.12^*^	19.57	22.18^*^
‘Mable Bell’	0	0.29	7.27	3.31	1.48	2.04	0.67	15.11	8.43	36.07	17.36	19.90
	50	0.11^*^	9.45^*^	4.52^*^	2.20^*^	3.10^*^	0.81^*^	15.31	7.11^*^	51.09^*^	27.85^*^	25.58^*^
‘Pink Bell’	0	0.09	5.63	2.91	1.13	1.97	0.41	9.57	10.45	29.82	10.90	17.98
	50	0.11^*^	5.30^*^	3.28^*^	1.50^*^	1.86	0.48^*^	10.86^*^	9.14^*^	36.12^*^	18.49^*^	18.65
‘Peach Bowl’	0	0.08	4.79	2.99	1.11	1.50	0.43	7.98	8.06	37.35	11.91	19.59
	50	0.11^*^	6.58^*^	4.02^*^	1.40^*^	2.18^*^	0.57^*^	9.56^*^	11.43^*^	42.74^*^	20.68^*^	23.24^*^
*F*-test	A	***^y^	***	***	***	***	***	***	***	***	***	***
	B	***	***	***	***	***	***	***	NS	***	***	***
	A × B	***	***	***	***	***	***	***	***	***	***	***

Data were represented by the mean. Each treatment was measured three times. ^z^Asterisks represent significant differences between the control and the Si treatment according to Student’s *t*-test (*P* ≤ 0.05). ^y^NS, *, **, *** Non-significant or significant at *P* ≤ 0.05, 0.01, 0.001, respectively.
